# Investigation of the profile of phenolic compounds in the leaves and stems of *Pandiaka heudelotii* using gas chromatography coupled with flame ionization detector

**DOI:** 10.1002/fsn3.443

**Published:** 2016-11-23

**Authors:** Mercy O. Ifeanacho, Catherine C. Ikewuchi, Jude C. Ikewuchi

**Affiliations:** ^1^ Department of BiochemistryFaculty of ScienceUniversity of Port HarcourtPort HarcourtNigeria

**Keywords:** flavonoids, hydroxycinnamates, lignans, *Pandiaka heudelotii*, phenolic acids

## Abstract

The profile of phenolic compounds in the leaves and stems of *Pandiaka heudelotii* was investigated using gas chromatography coupled with flame ionization detector. The leaves and stems had high flavonoids and benzoic acid derivatives content, and moderate levels of lignans and hydroxycinnamates. Twenty‐eight known flavonoids were detected, which consisted mainly of kaempferol (41.93% in leaves and 47.97% in stems), (+)‐catechin (17.12% in leaves and 16.11% in stems), quercetin (13.83% in leaves and 9.39% in stems), luteolin (7.34% in leaves and 7.71% in stems), and artemetin (6.53% in leaves and 4.83% in stems). Of the six known hydroxycinnamates detected, chlorogenic acid (80.79% in leaves and 87.56% in stems) and caffeic acid (18.98% in leaves and 12.30% in stems) were the most abundant, while arctigenin (77.81% in leaves and 83.40% in stems) and retusin (13.82% in leaves and 10.59% in stems) were the most abundant of the nine known lignans detected. Twelve known benzoic acid derivatives were detected, consisting mainly of ellagic acid (65.44% in leaves and 72.89% in stems), p‐hydroxybenzoic acid (25.10% in leaves and 18.95% in stems), and vanillic acid (8.80% in leaves and 7.30% in stems). The rich phytochemical profile of the leaves and stems is an indication of their ability to serve as sources of nutraceuticals.

## 1 Introduction

The wide use of phytonutrients reflects a fact that nutrition science has advanced beyond the treatment of deficiency syndromes to the reduction of disease risk. Food is no longer evaluated only in terms of macronutrient and micronutrient levels, but their contents of some biologically active compounds are becoming more important (Zhao, 2007). In addition to providing macro‐ and micro‐nutrients, vegetables are rich sources of bioactive phytochemicals, and other compounds that support human health and nutrition (Radovich, 2011; Sinha, Hui, Evranuz, Siddiq, & Ahmed, 2011). *Pandiaka heudelotii* (family: Amaranthaceae) is a wild vegetable commonly consumed in southern Nigeria. It is used for the preparation of soup, and boiled for tea. Despite the use of this plant as both food and medicine, we found no information in the biochemical literature regarding its phytochemical and phenolic compounds composition. Therefore, this study investigated the phenolic compounds composition of the leaves and stems of *Pandiaka heudelotii* with a view to providing information on their potential as sources of nutraceuticals.

## 2 Materials and Methods

### 2.1 Collection of samples

Fresh samples of *Pandiaka heudelotii* were collected from within the Abuja Campus of University of Port Harcourt, Port Harcourt, Nigeria. They were duly identified at the Herbarium of the Department of Plant Science and Biotechnology, University of Port Harcourt. The leaves were collected and rid of dirt, and used for the analysis.

### 2.2 General procedures

Gas chromatography was carried out at Multi Environmental Management Consultants Limited, Igbe Road, Ikorodu, Lagos, with a Hewlett Packard HP 6890, gas chromatograph, fitted with a flame ionization detector (FID), and powered with HP Chemstation Rev. A09.01[1206] software, to identify and quantify compounds. Standards were from Sigma‐Aldrich Co. and Lynnchem Biological Technology Co. Standard solutions were prepared in methanol for flavonoids and benzoic acid derivatives, acetone for lignans, and ethanol for hydroxycinnamates. The linearity of the dependence of response on concentration was verified by regression analysis. Identification was based on comparison of retention times and spectral data of the standards. Quantification was performed by establishing calibration curves for each compound determined, using the standards.

### 2.3 Determination of benzoic acid derivatives composition

Benzoic acid derivatives were extracted by the two‐stage process described by Andary et al. (2013). The concentrated extract (2.0 ml) was transferred to a 5.0 ml glass vial. It was then saturated with sodium chloride salt before adding 250.0 μl of ethyl acetate to it. The mixture was agitated manually for 10 min at room temperature and later centrifuged for 15 min at 2500 rpm, before removing the organic phase to a 1‐ml vial. The extraction was repeated twice and the organic phases were combined. Aliquot of 50.0 μl of N,O‐bis (trimethylsilyl) trifluoroacetamide was added, and the mixture was manually agitated for 2 min at room temperature for derivatization. The column type was a capillary HP‐1 with the dimension (30 m × 0.25 mm × 0.25 μm film thickness). The inlet and detector temperatures were 250°C and 320°C, respectively. Split injection was adopted with a split ratio of 20:1. Nitrogen was used as the carrier gas. The hydrogen and compressed air pressures were 193.1 kPa and 220.6 kPa. The oven was programmed as follows: initial temperature at 60°C for 5 min, ramping at 15°C/min for 15 min, and attained temperature maintained for 1 min, followed by a second ramping at 10°C/min for 4 min.

### 2.4 Determination of the flavonoids composition

The flavonoids extraction was carried out according to the method of Millogo‐Kone et al. (2009). One gram of the dried ethanol and aqueous extracts were weighed into 100 ml of distilled water in a 250‐ml conical flask and boiled for 10 min. To this was added 100 ml of boiling methanol/water (70:30, v:v) mixture. The mixture was allowed to macerate for about 2 hr, and then filtered with Whatman filter paper No.1. The filtrate was concentrated to 5 ml for gas chromatographic analysis. The column was a capillary HP INNOWax (30 m × 0.25 mm × 0.25 μm film thickness). The inlet and detection temperatures were 250 and 320°C. Split injection was adopted with a split ratio of 20:1. Nitrogen was used as the carrier gas. The hydrogen and compressed air pressures were 151.7 kPa and 241.3 kPa. The oven was programmed as follows: initial temperature of 50°C, first ramping at 8°C/min for 20 min, attained temperature maintained for 4 min, followed by a second ramping at 12°C/min for 4 min, and maintaining attained temperature for 4 min.

### 2.5 Determination of hydroxycinnamates composition

The hydroxycinnamates extract was prepared as described by Ortan, Popescu, Gaita, Dinu‐Pîrvu, and Câmpeanu (2009), and subjected to gas chromatographic analysis. The column was HP‐5 (30 m × 0.32 mm × 0.25 μm film thickness). The samples were introduced via an all‐glass injector working in the split mode, with nitrogen as the carrier gas, at a flow rate of 1 ml/min. The injection and detector temperatures were 260°C and 300°C, respectively. The oven temperature was programmed at the start of the run from 170°C to 250°C at 5°C/min.

### 2.6 Determination of the lignans composition

The lignan extract was prepared as reported by Chapman, Knoy, Kindscher, Brown, and Niemann (2006), and subjected to gas chromatography. The column was ZP‐5 (30 m × 0.32 mm × 0.25 μm film thickness), detected at 300 nm. One microliter of sample was injected. The conditions for the GC were initial oven temperature of 40°C, injector 250°C, transfer line 280°C, a solvent delay of 2 min; and ramped temperature at 10°C/min to a final temperature of 230°C, which was held for 1 min.

### 2.7 Determination of tannins composition

The tannin extract was prepared as reported by Luthar (1992), and subjected to gas chromatographic analysis. The column was a capillary HP‐5 (30 m × 0.25 mm × 0.25 μm film thickness). The inlet and detection temperatures were 250 and 320°C. Split injection was adopted with a split ratio of 20:1. Nitrogen was used as the carrier gas. The hydrogen and compressed air pressures were 173.1 kPa and 275.8 kPa. The oven was programmed to an initial temperature at 120°C, before ramping at 10°C/min for 20 min.

## 3 Results

The leaves (5885.5 mg/kg dry weight) and stems (3644.0 mg/kg dry weight) had high total flavonoid contents (Table 1). Twenty‐eight known flavonoids were detected, which consisted of kaempferol (41.9% in leaves and 48.0% in stems), (+)‐catechin (17.1% in leaves and 16.1% in stems), quercetin (13.8% in leaves and 9.4% in stems), luteolin (7.3% in leaves and 7.7% in stems), artemetin (6.5% in leaves and 4.8% in stems), naringin (6.1% in leaves and 4.7% in stems), apigenin (4.0% in leaves and 4.7% in stems), hesperidin (3.1% in leaves and 2.4% in stems) all comprising above 99%. The remainder (less than 1%) consisted of isorhamnetin, naringenin, myricetin, (−)‐epicatechin, daidzein, genistein, biochanin, silymarin, daidzin, gallocatechin, butein, robinetin, tangeretin, baicalein, resveratrol, baicalin, nobiletin, (−)‐epicatechin‐3‐gallate, (−)‐epigallocatechin, and (−)‐epigallocatechin‐3‐gallate.

**Table 1:** Isolated and detected flavonoids in the leaves and stems of *Pandiaka heudelotii*

Compounds	Composition (mg/kg)					
	Leaves			Stems		
	Retention time (min)	Fresh weight	Dry weight	Retention time (min)	Fresh weight	Dry weight
(+)‐Catechin	13.696	890	1000	13.698	540	590
Apigenin	14.509	210	240	14.509	160	170
Resveratrol	15.105	0.00027	0.00031	15.103	0.00015	0.00016
Genistein	15.537	0.00071	0.00080	15.536	0.00037	0.00040
Daidzein	15.805	0.00072	0.00082	15.805	0.00036	0.00039
Daidzin	16.254	0.00052	0.00059	16.254	0.00027	0.00030
Butein	16.539	0.00038	0.00043	16.542	0.00020	0.00022
Naringenin	16.926	0.014	0.016	16.925	0.0072	0.0078
Biochanin	17.205	0.00067	0.00076	17.206	0.00035	0.00039
Luteolin	17.451	380	430	17.451	260	280
Kaempferol	17.711	2200	2500	17.706	1600	1700
(−)‐Epicatechin	18.784	0.0014	0.0016	18.782	69	75
(−)‐Epigallocatechin	20.677	0.000062	0.000071	20.450	0.00017	0.00019
Quercetin	21.094	720	810	21.209	310	340
Gallocatechin	22.577	0.00044	0.00050	22.578	0.00010	0.00011
(−)‐Epicatechin‐3‐gallate	22.858	0.00015	0.00017	22.856	0.000073	0.000079
(−)‐Epigallocatechin‐3‐gallate	23.575	0.00005	0.00006	23.573	0.000024	0.000026
Isorhamnetin	24.037	0.27	0.30	24.038	0.12	0.13
Robinetin	24.154	0.00033	0.00037	24.153	0.00016	0.00018
Myricetin	24.714	0.0014	0.0016	24.787	0.00031	0.00033
Baicalein	25.564	0.00032	0.00036	25.557	0.00016	0.00018
Nobiletin	26.177	0.00016	0.00019	26.177	0.00008	0.00009
Baicalin	26.381	0.00027	0.00030	26.381	0.00014	0.00015
Tangeretin	26.529	0.00032	0.00036	26.530	0.00015	0.00017
Artemetin	26.741	340	380	26.742	160	180
Silymarin	27.085	0.00063	0.00071	27.084	0.00033	0.00036
Naringin	27.490	320	360	27.489	160	170
Hesperidin	28.321	160	180	28.315	81	89
Total flavonoids content		5200	5900		3300	3600

The total benzoic acid derivatives’ contents of the leaves and stems of *Pandiaka heudelotii* were 3384.1 mg/kg dw and 1975.4 mg/kg dw, respectively (Table 2). Twelve known compounds were detected including ellagic acid (65.4% in leaves and 72.9% in stems), p‐hydroxybenzoic acid (25.1% in leaves and 19.0% in stems), vanillic acid (8.8% in leaves and 7.3% in stems), and rosmarinic acid (0.6% in leaves and 0.8% in stems); comprising above 99%. The remainder consisted of syringic acid, ferulic acid, sinapinic acid, o‐coumaric acid, piperic acid, gentisic acid, protocatechuic acid, and cinnamic acid.

**Table 2:** Isolated and detected benzoic acid derivatives in the leaves and stems of *Pandiaka heudelotii*

Compounds	Composition (mg/kg)					
	Leaves			Stems		
	Retention time (min)	Fresh weight	Dry weight	Retention time (min)	Fresh weight	Dry weight
Cinnamic acid	9.325	0.0025	0.0029	9.008	0.000064	0.000069
Gentisic acid	10.848	0.0044	0.0050	10.844	0.000072	0.000079
Protocatechuic acid	12.357	0.0021	0.0024	12.367	0.00017	0.00018
Vanillic acid	14.918	260	300	14.917	130	140
o‐Coumaric acid	15.812	0.082	0.093	15.815	0.030	0.033
p‐Hydroxybenzoic acid	16.037	750	850	16.038	340	370
Ferulic acid	18.493	0.21	0.24	18.498	0.14	0.15
Syringic acid	19.688	1.1	1.2	19.626	0.42	0.46
Piperic acid	20.467	0.0064	0.0073	20.473	0.0049	0.0053
Sinapinic acid	21.327	0.092	0.10	21.329	0.090	0.098
Ellagic acid	22.602	1900	2200	22.606	1300	1400
Rosmarinic acid	23.232	18	21	23.235	15	16
Total phenolic acids content		3000	3400		1800	2000

They had moderate total (448.4 mg/kg dw in leaves and 334.3 mg/kg dw in stems) hydroxycinnamates’ contents (Table 3). Six known hydroxycinnamates were detected, including chlorogenic acid (80.87% in leaves and 87.6% in stems), caffeic acid (19.0% in leaves and 12.3% in stems), and chicoric acid (0.1% in leaves and 0.1% in stems). The remaining less than 0.2% consisted of p‐coumaric acid, p‐coumarin, and scopoletin. Tannic acid was the only compound detected in the tannins fraction.

**Table 3:** Isolated and detected hydroxycinnamates and tannins in the leaves and stems of *Pandiaka heudelotii*

Compounds	Composition (mg/kg)					
	Leaves			Stems		
	Retention time (min)	Fresh weight	Dry weight	Retention time (min)	Fresh weight	Dry weight
Hydroxycinnamates						
p‐Coumarin	7.790	0.083	0.094	7.793	0.061	0.066
p‐Coumaric acid	11.522	0.34	0.38	11.528	0.15	0.17
Caffeic acid	14.400	75	85	14.397	38	41
Scopoletin	16.335	0.11	0.12	16.365	0.030	0.033
Chlorogenic acid	19.030	320	360	19.016	270	290
Chicoric acid	20.349	0.40	0.45	20.341	0.18	0.20
Total hydroxycinnamates content		390	450		310	330
Tannins						
Tannic acid	19.518	50	57	19.518	51	56
Total tannins content		50	57		51	56

The total lignans contents of the leaves and stems were 496.6 mg/kg dw and 914.6 mg/kg dw, respectively (Table 4). Nine known lignans were detected, consisting of arctigenin (77.8% in leaves and 83.4% in stems), retusin (13.8% in leaves and 10.6% in stems), dehydroabietic acid (8.1% in leaves and 5.8% in stems), and sakuranin (0.2% in leaves and 0.1% in stems). The remaining less than 0.2% consisted of epieudesmin, galgravin, apigenin‐4′,7‐dimethyl ether, 2‐allyl‐5‐ethoxy‐4‐methoxyphenol, and (9E, 12E, 15E)‐9,12,15‐octadecatrien‐1‐ol.

**Table 4:** Isolated and detected lignans in the leaves and stems of *Pandiaka heudelotii*

Compounds	Composition (mg/kg)					
	Leaves			Stems		
	Retention time (min)	Fresh weight	Dry weight	Retention time (min)	Fresh weight	Dry weight
2‐Allyl‐5‐ethoxy‐4‐methoxyphenol	11.449	0.0034	0.0039	11.298	0.0066	0.00720
(9E, 12E, 15E)‐9,12,15‐Octadecatrien‐1‐ol	14.143	0.00021	0.00024	14.130	0.00010	0.00011
Apigenin‐4′,7‐dimethyl ether	16.372	0.048	0.054	16.429	0.049	0.053
Dehydroabietic acid	18.133	35	40	18.551	49	53
Retusin	19.675	61	69	20.008	89	97
Galgravin	20.463	0.13	0.15	20.320	0.090	0.098
Arctigenin	21.131	340	39	21.393	700	760
Epieudesmin	22.360	0.24	0.27	22.351	0.32	0.35
Sakuranin	23.968	0.78	0.89	23.963	1.2	1.3
Total lignans content		440	50		840	910

## 4 Discussion

This study showed that Pandiaka leaves have higher artemetin contents than *Artemisia annua* (Weathers & Towler, 2012). Artemetin has anti‐inflammatory (Sertie, Basile, Panizza, Matida, & Zelnik, 1990), antiedematogenic (Bayeux, Fernandes, Foglio, & Carvalho, 2002), antioxidant (Dugas et al., 2000), and hypotensive properties (de Souza et al., 2011). The amygdalin content of the leaves was higher than that of sweet (non‐bitter) almonds, but lower than those of slightly bitter (semi‐bitter) almond and apricots (Lee, Zhang, Wood, Castillo, & Mitchell, 2013; Yildirim & Askin, 2010). Amygdalin (D‐mandelonitrile‐b‐D‐gentiobioside) has antitussive, lubricant, and antitumor activities (Lv, Ding, & Zheng, 2005; Syrigos, Rowlinson, & Epenetos, 1998). It is decomposed by the action of b‐D‐glucosidase to yield hydrocyanic acid, which reflexively stimulates the respiratory center and produces antitussive and antiasthmatic effects (Isozaki, Matano, Yamamoto, Kosaka, & Tani, 2001; Lv et al., 2005).

When compared to blueberry, the leaves and stems had higher quercetin (73 mg/kg, US Highbush Blueberry Council, 2005; 17–24 mg/kg, Hakkinen, Karenlampi, Heinonen, Mykkanen, & Torronen, 1999), while the stems had higher epicatechin (11.1 mg/kg, US Highbush Blueberry Council, 2005) contents. Pandiaka leaves and stems also had higher apigenin, catechin, hesperidin, luteolin, kaempferol, and quercetin contents than lettuce, onions, and carrots (Harnly et al., 2006). The leaves and stems were also rich in arctigenin, retusin, ellagic, p‐hydroxybenzoic, vanillic, chlorogenic, caffeic, tannic, and dehydroabietic acids. This means that they can serve as sources of these bioactive compounds.

Studies have shown that kaempferol, apigenin, quercetin, catechin, and luteolin have antioxidant, antidiabetic, hypolipidemic, hypotensive, antibacterial, anti‐inflammatory, and anticancer properties (Dillard & German, 2000; Sutherland, Rahman, & Appleton, 2006; Lakhanpal & Rai, 2007; Panda & Kar, 2007; Calderón‐Montaño, Burgos‐Morón, Pérez‐Guerrero, & López‐Lázaro, 2011; Ren et al., 2016). Other properties of kaempferol include analgesic, antiallergic, antiprotozoal, antiviral, antifungal, neuroprotective, cardio‐protective, and hepatoprotective properties (Calderón‐Montaño et al., 2011; Dillard & German, 2000); while those of apigenin are diuretic, hepatoprotective, and cardio‐protective properties (Dillard & German, 2000; Panda & Kar, 2007; Ren et al., 2016). In addition to the above, catechin also have antiviral, antiallergic, antiobesity, antiplatelet, antiulcer, chemo‐preventive, neuroprotective, cardio‐protective, antispasmodic, bronchodilator, and vasodilator properties (Dillard & German, 2000; Ghayur, Khan, & Gilani, 2007; Sutherland et al., 2006); while quercetin has antiallergic, antiarthritic, anticataractogenic, antiviral, cardio‐protective, gastro‐protective, and hepatoprotective activities (Dillard & German, 2000; Lakhanpal & Rai, 2007). Luteolin also has antiallergic, antiandrogenic, antiestrogenic, neuroprotective, and radio‐protective activities (Dillard & German, 2000; López‐Lázaro, 2009).

Vanillic and 4‐hydroxybenzoic acids have antifungal, antimutagenic, antisickling, estrogenic, and antimicrobial activities (Khadem & Marles, 2010; Oksana, Marian, Mahendra, & Bo, 2012). In addition, vanillic acid is a flavoring, anthelmintic, hepatoprotective, immune‐modulating, and anti‐inflammatory agent (Khadem & Marles, 2010; Oksana et al., 2012). Ellagic acid is reported to have antioxidant, antimalarial, anti‐inflammatory, antiallergic, antidiabetic, antiatherogenic, antiwrinkle, antidepressant, neuroprotective, antiapoptotic, anticancer, antiproliferative, and chemo‐preventive activities (Dhingra & Chhillar, 2012; Özkaya et al., 2013).

Chlorogenic acid reduces the risk of cardiovascular disease, and exhibits many biological properties such as antibacterial, antiviral, anti‐inflammatory, antioxidant, anticancer, antiobesity, hypolipidemic, hepatoprotective, immunostimulatory, hypoglycemic, and antihypertensive activities (Cho et al., 2010; Farah, 2012; Lafay, Morand, Manach, Besson, & Scalbert, 2006; Li, Habasi, Xie, & Aisa, 2014; Meng, Cao, Feng, Peng, & Hu, 2013; Zhao, Wang, Ballevre, Luo, & Zhang, 2011). Caffeic acid increases collagen production, in addition to having antiaging, antioxidant, antimicrobial, antiatherosclerotic, antidiabetic, antitumor, anti‐inflammatory, and photo‐protective properties (Dhingra & Chhillar, 2012; Gugliucci, Bastos, Schulze, & Souza, 2009; Magnani, Isaac, Correa, & Salgado, 2014; Oksana et al., 2012). Studies have shown that arctigenin has antioxidant, antitumor, anti‐inflammatory, antiviral, analgesic, neuroprotective, and memory‐enhancing activities (Chakraborty & Borah, 2013; Du et al., 2016; Lu et al., 2015; Park, Hong, Moon, Kim, & Kim, 2011; Srivastava & Shukla, 2015; Zhu et al., 2013). Retusin is an antiemetic, antitumor, and psychoactive agent; and teas containing it are used as anti‐inflammatory agents, analgesics, purgative, laxative, and cathartic (Chakrapani et al., 2013; Chapman et al., 2006).

From the foregoing, it can be seen that the leaves and stems of *Pandiaka heudelotii* contain a variety of biologically active phytochemicals. The beneficial roles of these bioactive phytochemical constituents can be harnessed in the diet, making them important tools for nutritional therapy. This, therefore, emphasizes the potential of the leaves as a candidate for use as functional food.

## Conflict of Interest

None declared.

## References

[fsn3443-bib-0001] Andary, J. , Maalouly, J. , Ouaini, R. , Chebib, H. , Beyrouthy, M. , Rutledge, N. D. , & Ouaini, N. (2013). Phenolic compounds from diluted acid hydrolysates of olive stone: Effects of over‐liming. Advances in Crop Science and Technology, 1, 1.

[fsn3443-bib-0002] Bayeux, M. C. , Fernandes, A. T. , Foglio, M. A. , & Carvalho, J. E. (2002). Evaluation of the antiedematogenic activity of artemetin isolated from *Cordia curassavica* DC. Brazilian Journal of Medical and Biological Research, 35, 1229–1232. 12424497 10.1590/s0100-879x2002001000017

[fsn3443-bib-0003] Calderón‐Montaño, J. M. , Burgos‐Morón, E. , Pérez‐Guerrero, C. , & López‐Lázaro, M. (2011). A review on the dietary flavonoid kaempferol. Mini‐Reviews in Medicinal Chemistry, 11, 298–344. doi:10.2174/138955711795305335 21428901 10.2174/138955711795305335

[fsn3443-bib-0004] Chakraborty, D. , & Borah, S. (2013). Japanese encephalitis: An overview of the disease with special reference to typical therapeutic measures. International Research Journal of Pharmaceutical and Applied Sciences, 3(2), 130–136.

[fsn3443-bib-0005] Chakrapani, P. , Venkatesh, K. , Chandra, S. S. B. , Arun, J. B. , Prem, K. , Amareshwari, P. , & Rani, A. R. (2013). Phytochemical, pharmacological importance of patchouli (Pogostemon cablin (Blanco) Benth) an aromatic medicinal plant. International Journal of Pharmaceutical Sciences Review and Research, 21(2), 7–15.

[fsn3443-bib-0006] Chapman, J. M. , Knoy, C. , Kindscher, K. , Brown, R. C. D. , & Niemann, S . (2006). Identification of antineoplastic and neurotrophic lignans in medicinal Prairie plants by liquid chromatography electron impact mass spectrometry (LC/EI/MS). Reprint of Poster from Kansas City Life Sciences Day 2006. Retrieved from: http://www.cssco.com/files/KC%20Life%20Science%202006.pdf [last accessed 10 September 2009].

[fsn3443-bib-0007] Cho, A.‐S. , Jeon, S.‐M. , Kim, M.‐J. , Yeo, J. , Seo, K.‐I. , Choi, M.‐S. , & Lee, M.‐K. (2010). Chlorogenic acid exhibits anti‐obesity property and improves lipid metabolism in high‐fat diet‐induced‐obese mice. Food and Chemical Toxicology, 48, 937–943. 20064576 10.1016/j.fct.2010.01.003

[fsn3443-bib-0008] de Souza, P. , Gasparotto, A. Jr , Crestania, S. , Stefanello, M. É. A. , Marques, M. C. A. , da Silva‐Santos, J. E. , & Kassuya, C. A. L. (2011). Hypotensive mechanism of the extracts and artemetin isolated from *Achillea millefolium* L. (Asteraceae) in rats. Phytomedicine, 18, 819–825. 21420289 10.1016/j.phymed.2011.02.005

[fsn3443-bib-0009] Dhingra, D. , & Chhillar, R. , (2012). Antidepressant‐like activity of ellagic acid in unstressed and acute immobilization‐induced stressed mice. Pharmacological Reports, 64, 796–807. Available from http://www.if-pan.krakow.pl/pjp/pdf/2012/4_796.pdf doi:10.1016/S1734‐1140(12)70875‐7 23087132 10.1016/s1734-1140(12)70875-7

[fsn3443-bib-0010] Dillard, C. J. , & German, J. B. (2000). Phytochemicals: Nutraceuticals and human health. Journal of the Science of Food and Agriculture, 80, 1744–1756. doi:10.1002/1097‐0010(20000915)80:12<1744:AID‐JSFA725>3.0.CO;2‐W

[fsn3443-bib-0011] Du, Z.‐C. , Xue, T. , Jiang, M. , Lu, H.‐Y. , Ye, Z.‐C. , Ruan, B.‐J. , … Wang, L. (2016). Arctigenin attenuates imiquimod‐induced psoriasis‐like skin lesions via down‐regulating keratin17. International Journal of Clinical and Experimental Medicine, 9(2), 1639–1647.

[fsn3443-bib-0012] Dugas, A. J. Jr , Castaneda‐Acosta, J. , Bonin, G. C. , Price, K. L. , Fischer, N. H. , & Winston, G. W. (2000). Evaluation of the total peroxyl radical‐scavenging capacity of flavonoids: Structure–activity relationships. Journal of Natural Products, 63, 327–331. 10757712 10.1021/np990352n

[fsn3443-bib-0013] Farah, A. (2012). Coffee constituents In Chu Y.‐F. (Ed.), Coffee: Emerging health effects and disease prevention, 1st ed. USA: Blackwell Publishing Ltd.

[fsn3443-bib-0014] Ghayur, M. N. , Khan, H. , & Gilani, A. H. (2007). Antispasmodic, bronchodilator and vasodilator activities of (+)‐catechin, a naturally occurring flavonoid. Archives of Pharmacal Research, 30, 970–975. doi:10.1007/BF02993965 17879750 10.1007/BF02993965

[fsn3443-bib-0015] Gugliucci, A. , Bastos, D. H. M. , Schulze, J. , & Souza, M. F. F. (2009). Caffeic and chlorogenic acids in *Ilex paraguariensis* extracts are the main inhibitors of AGE generation by methylglyoxal in model proteins. Fitoterapia, 80, 339–344. doi:10.1016/j.fitote.2009.04.007 19409454 10.1016/j.fitote.2009.04.007

[fsn3443-bib-0016] Hakkinen, S. H. , Karenlampi, S. O. , Heinonen, I. M. , Mykkanen, H. M. , & Torronen, A. R. (1999). Content of the flavanols quercetin, myricetin, and kaempferol in 25 edible berries. Journal of Agricultural and Food Chemistry, 47, 2274–2279. 10794622 10.1021/jf9811065

[fsn3443-bib-0017] Harnly, J. M. , Doherty, R. F. , Beecher, G. R. , Holden, J. M. , Haytowitz, D. B. , Bhagwat, S. , & Gebhardt, S. (2006). Flavonoid Content of U.S. Fruits, Vegetables, and Nuts. Journal of Agriculture and Food Chemistry, 54(26), 9966–9977. doi:10.1021/jf061478a 10.1021/jf061478a 17177529

[fsn3443-bib-0018] Isozaki, T. , Matano, Y. , Yamamoto, K. , Kosaka, N. , & Tani, T. (2001). Quantitative determination of amygdalin epimers by cyclodextrin‐modified micellar electrokinetic chromatography. Journal of Chromatography A, 923, 249–254. 11510547 10.1016/s0021-9673(01)00969-4

[fsn3443-bib-0019] Khadem, S. , & Marles, R. J. (2010). Monocyclic phenolic acids; hydroxy‐ and polyhydroxybenzoic acids: Occurrence and recent bioactivity studies. Molecules, 15, 7985–8005. doi:10.3390/molecules15117985 21060304 10.3390/molecules15117985 PMC6259451

[fsn3443-bib-0020] Lafay, S. , Morand, C. , Manach, C. , Besson, C. , & Scalbert, A. (2006). Absorption and metabolism of caffeic acid and chlorogenic acid in the small intestine of rats. British Journal of Nutrition, 96, 39–46. doi:10.1079/BJN20051714 16869989 10.1079/bjn20061714

[fsn3443-bib-0021] Lakhanpal, P. , & Rai, D. K . (2007). Quercetin: A versatile flavonoid. Internet Journal of Medical Update, 2, 22–37. Available from http://www.akspublication.com/Paper05Jul-Dec2007.pdf

[fsn3443-bib-0022] Lee, J. , Zhang, G. , Wood, E. , Castillo, C. R. , & Mitchell, A. E. (2013). Quantification of amygdalin in non‐bitter, semi‐bitter, and bitter almonds (*Prunus dulcis*) by UHPLC‐(ESI)QqQ MS/MS. Journal of Agricultural and Food Chemistry, 61, 7754–7759. 23862656 10.1021/jf402295u

[fsn3443-bib-0023] Li, H.‐R. , Habasi, M. , Xie, L.‐Z. , & Aisa, H. A. (2014). Effect of chlorogenic acid on melanogenesis of B16 melanoma cells. Molecules, 19, 12940–12948. doi:10.3390/molecules190912940 25157464 10.3390/molecules190912940 PMC6271456

[fsn3443-bib-0024] López‐Lázaro, M. (2009). Distribution and biological activities of the flavonoid luteolin. Mini‐Reviews in Medicinal Chemistry, 9, 31–59. doi:10.2174/138955709787001712 19149659 10.2174/138955709787001712

[fsn3443-bib-0025] Lu, Z. , Cao, S. , Zhou, H. , Hua, L. , Zhang, S. , & Cao, J. (2015). Mechanism of arctigenin‐induced specific cytotoxicity against human hepatocellular carcinoma cell lines: Hep G2 and SMMC7721. PLoS ONE, 10(5), e0125727. doi:10.1371/journal.pone.0125727 25933104 10.1371/journal.pone.0125727 PMC4416797

[fsn3443-bib-0026] Luthar, Z . (1992). Polyphenol classification and tannin content of buckwheat seeds (*Fagopyrum esculentum* Moench). Fagopyrum, 12, 36–42. Available from: http://fagopyrum.uhostall.com/Fagopyrum/Fagopyrum/Each/Fag(12)/Fag(12)-36.pdf

[fsn3443-bib-0027] Lv, W.‐F. , Ding, M.‐Y. , & Zheng, R. (2005). Isolation and quantitation of amygdalin in apricot‐kernel and *Prunus tomentosa* Thunb. by HPLC with solid‐phase extraction. Journal of Chromatographic Science, 43, 383–387. 16176653 10.1093/chromsci/43.7.383

[fsn3443-bib-0028] Magnani, C. , Isaac, V. L. B. , Correa, M. A. , & Salgado, H. R. N. (2014). Caffeic acid: A review of its potential use in medications and cosmetics. Analytical Methods, 6, 3203–3210. doi:10.1039/c3ay41807c

[fsn3443-bib-0029] Meng, S. , Cao, J. , Feng, Q. , Peng, J. , & Hu, Y . (2013). Roles of chlorogenic acid on regulating glucose and lipids metabolism: A review. Evidence‐Based Complementary and Alternative Medicine, 2013, Article ID 801457, 11 pages. doi: org/10.1155/2013/801457 24062792 10.1155/2013/801457 PMC3766985

[fsn3443-bib-0030] Millogo‐Kone, H. , Lompo, M. , Kini, F. , Asimi, S. , Guissou, I. P. , & Nacoulma, O. (2009). Evaluation of flavonoids and total phenolic contents of stem bark and leaves of *Parkia biglobosa* (Jacq.) Benth. (Mimosaceae)‐free radical scavenging and antimicrobial activities. *Research Journal of* . Medical Science, 3(2), 70–74.

[fsn3443-bib-0031] Oksana, S. , Marian, B. , Mahendra, R. , & Bo, S. H. (2012). Plant phenolic compounds for food, pharmaceutical and cosmetics production. Journal of Medicinal Plants Research, 6, 2526–2539.

[fsn3443-bib-0032] Ortan, A. , Popescu, M.‐L. , Gaita, A.‐L. , Dinu‐Pîrvu, C. , & Câmpeanu, G. H. (2009). Contributions to the pharmacognostical study on *Anethum graveolens*, Dill (Apiaceae). Romanian Biotechnology Letters, 14(2), 4342–4348.

[fsn3443-bib-0033] Özkaya, A. , Çiftçi, H. , Dayangaç, A. , Çevrimli, B. S. , Ölçücü, A. , & Çelik, S. (2013). Effects of ellagic acid and hesperetin on levels of some elements in livers of aluminum‐induced rats. Turkish Journal of Biochemistry, 38, 345–349. doi:10.5505/tjb.2013.29291

[fsn3443-bib-0034] Panda, S. , & Kar, A. (2007). Apigenin (4′,5,7‐trihydroxyflavone) regulates hyperglycaemia, thyroid dysfunction and lipid peroxidation in alloxan‐induced diabetic mice. Journal of Pharmacy and Pharmacology, 59, 1543–1548. doi:10.1211/jpp.59.11.0012 17976266 10.1211/jpp.59.11.0012

[fsn3443-bib-0035] Park, J.‐H. , Hong, Y.‐J. , Moon, E. , Kim, S.‐A. , & Kim, S. Y. (2011). *Forsythiae fructus* and its active component, arctigenin, provide neuroprotection by inhibiting neuroinflammation. Biomolecules and Therapeutics, 19(4), 425–430. doi:10.4062/biomolther.2011.19.4.425

[fsn3443-bib-0036] Radovich, T. J. K . (2011). Biology and classification of vegetables In Sinha N.K., Hui Y.H., Evranuz E. O., Siddiq M. & Ahmed J. (Eds.), Handbook of vegetables and vegetable processing (pp. 1–22). Iowa, USA: Blackwell Publishing Ltd.

[fsn3443-bib-0037] Ren, B. , Qin, W. , Wu, F. , Wang, S. , Pan, C. , Wang, L. , … Liang, J. (2016). Apigenin and naringenin regulate glucose and lipid metabolism, and ameliorate vascular dysfunction in type 2 diabetic rats. European Journal of Pharmacology, 773, 13–23. doi:10.1016/j.ejphar.2016.01.002 26801071 10.1016/j.ejphar.2016.01.002

[fsn3443-bib-0038] Sertie, J. A. , Basile, A. C. , Panizza, S. , Matida, A. K. , & Zelnik, R. (1990). Anti‐inflammatory activity and sub‐acute toxicity of artemetin. Planta Medica, 56, 36–40. 2356241 10.1055/s-2006-960879

[fsn3443-bib-0039] Sinha, N. K. , Hui, Y. H. , Evranuz, E. O. , Siddiq, M. , & Ahmed, J . (editors). (2011). Handbook of vegetables and vegetable processing. Iowa, USA: Blackwell Publishing Ltd.

[fsn3443-bib-0040] Srivastava, D. , & Shukla, K. (2015). *Ipomoea cairica*: A medicinal weed with promising health benefits. International Journal of Information Research and Review, 2(5), 687–694.

[fsn3443-bib-0041] Sutherland, B. A. , Rahman, R. M. A. , & Appleton, I. (2006). Mechanisms of action of green tea catechins, with a focus on ischemia‐induced neurodegeneration. Journal of Nutritional Biochemistry, 17, 291–306. doi:10.1016/j.jnutbio.2005.10.005 16443357 10.1016/j.jnutbio.2005.10.005

[fsn3443-bib-0042] Syrigos, K. N. , Rowlinson, B. G. , & Epenetos, A. A. (1998). In vitro cytotoxicity following specific activation of amygdalin by beta‐glucosidase conjugated to a bladder cancer‐associated monoclonal antibody. International Journal of Cancer, 78, 712–719. 9833764 10.1002/(sici)1097-0215(19981209)78:6<712::aid-ijc8>3.0.co;2-d

[fsn3443-bib-0043] US Highbush Blueberry Council , (2005). Composition of blueberries. Available from http://www.blueberry.org/Nutrition2.pdf [last accessed 15 March 2011].

[fsn3443-bib-0044] Weathers, P. J. , & Towler, M. J. (2012). The flavonoids casticin and artemetin are poorly extracted and are unstable in an *Artemisia annua* tea infusion. Planta Medica, 78(10), 1024–1026. doi:10.1055/s‐0032‐1314949 22673829 10.1055/s-0032-1314949 PMC3558976

[fsn3443-bib-0045] Yildirim, F. A. , & Askin, M. A. (2010). Variability of amygdalin content in seeds of sweet and bitter apricot cultivars in Turkey. African Journal of Biotechnology, 9(39), 6522–6524. doi:10.5897/AJB10.884

[fsn3443-bib-0046] Zhao, J. (2007). Nutraceuticals, nutritional therapy, phytonutrients, and phytotherapy for improvement of human health: A perspective on plant biotechnology application. Recent Patents in Biotechnology, 1, 75–97. 10.2174/187220807779813893 19075834

[fsn3443-bib-0047] Zhao, Y. , Wang, J. , Ballevre, O. , Luo, H. , & Zhang, W. (2011). Chlorogenic Antihypertensive effects and mechanisms of chlorogenic acids. Hypertension Research, 2011, 1–5. doi:10.1038/hr.2011.195 10.1038/hr.2011.195 22072103

[fsn3443-bib-0048] Zhu, Z. , Yan, J. , Jiang, W. , Yao, X.‐G. , Chen, J. , Chen, L. , … Shen, X. (2013). Arctigenin effectively ameliorates memory impairment in Alzheimer's disease model mice targeting both ‐amyloid production and clearance. Journal of Neuroscience, 33(32), 13138–13149. 23926267 10.1523/JNEUROSCI.4790-12.2013 PMC6619735

